# Identification of novel therapeutics for complex diseases from genome-wide association data

**DOI:** 10.1186/1755-8794-7-S1-S8

**Published:** 2014-05-08

**Authors:** Mani P Grover, Sara Ballouz, Kaavya A Mohanasundaram, Richard A George, Craig D H Sherman, Tamsyn M Crowley, Merridee A Wouters

**Affiliations:** 1 School of Medicine, Deakin University, Geelong, 3220, Victoria, Australia; 2 Cold Spring Harbor Laboratory, Cold Spring Harbor, NY 11724, USA; 3 Victor Chang Cardiac Research Institute, 405 Liverpool St, Darlinghurst, 2010, NSW, Australia; 4 Australian Animal Health Laboratory, CSIRO Animal, Food and Health Sciences, Portarlington Road, Geelong, 3219, Victoria, Australia; 5 Life and Environmental Sciences, Deakin University, Geelong, 3220, Victoria, Australia

**Keywords:** Complex disease, Candidate gene, Drug database, Drug target, Drug repositioning, Genome-wide association study

## Abstract

**Background:**

Human genome sequencing has enabled the association of phenotypes with genetic loci, but our ability to effectively translate this data to the clinic has not kept pace. Over the past 60 years, pharmaceutical companies have successfully demonstrated the safety and efficacy of over 1,200 novel therapeutic drugs via costly clinical studies. While this process must continue, better use can be made of the existing valuable data. *In silico *tools such as candidate gene prediction systems allow rapid identification of disease genes by identifying the most probable candidate genes linked to genetic markers of the disease or phenotype under investigation. Integration of drug-target data with candidate gene prediction systems can identify novel phenotypes which may benefit from current therapeutics. Such a drug repositioning tool can save valuable time and money spent on preclinical studies and phase I clinical trials.

**Methods:**

We previously used *Gentrepid *(http://www.gentrepid.org) as a platform to predict 1,497 candidate genes for the seven complex diseases considered in the Wellcome Trust Case-Control Consortium genome-wide association study; namely Type 2 Diabetes, Bipolar Disorder, Crohn's Disease, Hypertension, Type 1 Diabetes, Coronary Artery Disease and Rheumatoid Arthritis. Here, we adopted a simple approach to integrate drug data from three publicly available drug databases: the Therapeutic Target Database, the Pharmacogenomics Knowledgebase and DrugBank; with candidate gene predictions from *Gentrepid *at the systems level.

**Results:**

Using the publicly available drug databases as sources of drug-target association data, we identified a total of 428 candidate genes as novel therapeutic targets for the seven phenotypes of interest, and 2,130 drugs feasible for repositioning against the predicted novel targets.

**Conclusions:**

By integrating genetic, bioinformatic and drug data, we have demonstrated that currently available drugs may be repositioned as novel therapeutics for the seven diseases studied here, quickly taking advantage of prior work in pharmaceutics to translate ground-breaking results in genetics to clinical treatments.

## Background

The development of new therapeutics is essential to improve the human condition and lower the burden of disease. Due to our limited knowledge of the molecular basis of complex diseases, comparatively few gene targets for therapeutics have been identified to date. The standard approach to developing therapeutics involves testing many thousands of compounds against a known target in order to identify a lead compound. The lead compound can then be further refined *in silico *and *in vitro *before heading into the lengthy and costly clinical trials pipeline. This process, which consists of phases I, II, III and IV before final drug approval, involves 10-17 years of drug development, from target identification until FDA/EMEA approval, with only a 10% probability of success [1]. As a result, the pharmaceutical industry spends an average of about 1.2 billion US dollars to bring each new drug to market [2]. There is also a high risk associated with *de novo *drugs due to unforeseen adverse side effects, as seen in the case of Thalidomide, a drug used to treat morning sickness which resulted in devastating birth defects [3].

A novel approach to therapeutic development is to identify new applications for drugs that have already been approved, or have successfully completed phase I clinical trials which investigate toxicity [4,5]. This process of "drug repositioning" aims not to develop drugs *de novo*, but associate existing therapeutics with new phenotypes. Here, we attempted to reposition existing drugs to treat common complex diseases using recently acquired Genome-Wide Association Study (GWAS) data.

Complex diseases are genetically intricate, polygenic and multifactorial [6]; and frequently arise as a consequence of interaction between genes and the environment. Recently, GWAS have begun to unravel the complicated genetic basis of complex diseases. Sheer statistical power has allowed GWAS to successfully identify some associations between Single Nucleotide Polymorphisms (SNPs) and complex diseases [7]. Despite high investment, far fewer genes have been identified than can account for the heritable component of complex diseases, and the clinical benefit remains limited to date [8]. A factor that contributes to the missing heritability is likely to be noisy genotype-phenotype association signals [9]. Also, analysis of GWAS data using highly stringent thresholds for statistical significance, by testing multiple isolated SNPs, has limited the scope of gene discovery based on existing data [10]. As shown in Manhattan plots, GWAS data obviously contain far more information than the most significant peaks, and more work needs to be done extracting data from slightly less significant peaks [9,11].

Currently available gene discovery platforms can enhance candidate gene identification from GWAS data [9]. Candidate gene prediction tools are designed to find a needle in the genetic haystack. These tools are based on the assumption that genes with similar or related functions cause similar phenotypes [12]. Specific candidate gene prediction tools differ in the strategy adopted for calculating similarity, and the databases utilized for prediction [13,14]. *Gentrepid *is one of the many bioinformatic tools developed to help geneticists predict and prioritize candidate genes [9,15]. The *Gentrepid *tool and its knowledge base utilizes two independent methods: Common Pathway Scanning (CPS), a systems biology approach; and Common Module Profiling (CMP), a domain-based homology recognition approach, to prioritize candidate genes for human inherited disorders (see *Methods *for details). Compared to other prediction systems, *Gentrepid *is designed to make fewer, more conservative predictions which do not extensively extrapolate existing bioinformatic data i.e. it tends to be more specific than other systems [15].

We have previously developed protocols to analyze GWAS data using a multilocus approach which combines bioinformatic and genetic data [9,16,17]. To demonstrate the usefulness of these protocols, we reanalysed the well-studied Wellcome Trust Case-

Control Consortium (WTCCC) data for seven complex diseases [9]. Using a series of increasingly less conservative statistical thresholds, we attempted to discriminate the signal from the noise in the more statistically significant data (p ≤ 10-5, p ≤ 10-4, p ≤ 10-3). By incorporating bioinformatic data, we were able to predict 1,497 candidate genes for the seven complex diseases studied; namely, Type 2 Diabetes (T2D), Bipolar Disorder (BD), Crohn's Disease (CD), Hypertension (HT), Type 1 Diabetes (T1D), Coronary Artery Disease (CAD) and Rheumatoid Arthritis (RA) [9].

Here, we extend this pipeline to identify potential novel drug targets among the predicted candidate genes by associating drug information extracted from publicly available drug databases. The three databases sourced in this study were DrugBank [18], the Pharmacogenomics Knowledgebase (PharmGKB) [19] and the Therapeutic Target Database (TTD) [20]. The feasibility of this approach is again illustrated for the seven complex diseases investigated by the WTCCC [11]. This study shows that it is possible to identify therapeutics for treatment of specific complex diseases from genetic loci via the *Gentrepid *candidate gene prediction tool. Thus, in combination with drug target information, candidate gene prediction systems can be utilized as drug discovery tools to identify therapeutics which may be repositioned as novel treatments for complex diseases.

## Methods

**Figure 1:**
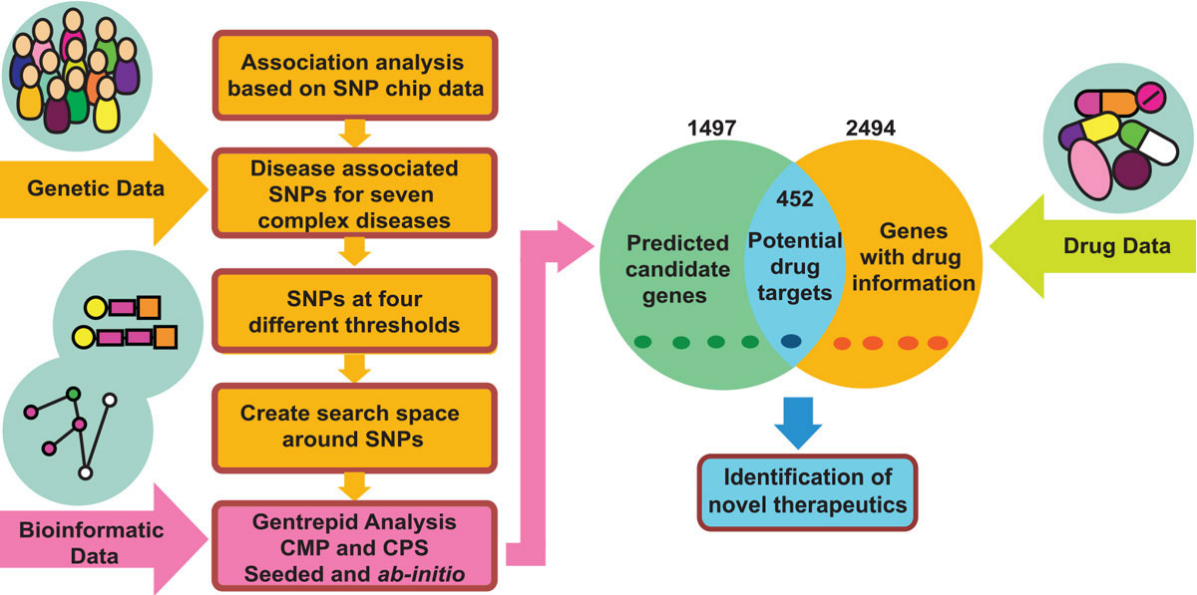
**Workflow**. The complete workflow designed to predict novel therapeutic targets and identify novel therapeutics. We used *Gentrepid *as a platform for candidate gene prediction and DrugBank, TTD and PharmGKB as drug repositories.

We implemented a computational workflow to enable repositioning of drugs by using *Gentrepid *as a bioinformatic candidate gene discovery platform, with drug data sourced from online databases (Figure 1). The two data sets integrated were:

1. A candidate gene data set obtained by integration of genotype-phenotype data from the WTCCC GWAS study on seven complex phenotypes [11], with bioinformatic data on structural domains and systems biology: identifying proteins that share common features, or participate in the same complex or pathway [21];

2. A drug-gene target association data set obtained from three drug databases namely TTD, DrugBank and PharmGKB [18-20].

## Candidate gene data set

In previous work, we predicted a total of 1,497 candidate genes for seven complex diseases by careful reanalysis of the WTCCC GWAS data [11] using the *Gentrepid *candidate gene prediction system [9].

In the original analysis, a highly stringent significance threshold (p ≤ 5x10^-7^) was used in an attempt to correct for multiple testing [11]. This conservative statistical approach, combined with the selection of the nearest-neighboring gene to the significant SNP, resulted in identification of only a small number of loci associated with each phenotype, with modest cumulative heritability [9] (Additional file 1, Table S1).

We specifically addressed these two issues in our reanalysis of this noisy data by - (a) Considering a series of four thresholds of decreasing stringency, starting with the highly significant threshold used in the original study, and decreasing to weakly significant(WS - p ≤ 10^-3^). This resulted in a series of four SNP sets containing up to 1064 SNPs being considered for each phenotype [9]. The number of loci and SNPs considered in the four data sets for each phenotype is shown in Table S1 (Additional file 1).

(b) Creating six different search spaces around each SNP-based locus, three of fixed-widths and three proximity-based, for analysis by our candidate gene prediction system [9].

Thus, for each of the seven phenotypes, twenty-four search spaces were constructed; using four SNP significance thresholds to obtain the loci, and six gene selection methods to construct the gene search spaces. In total, 168 search spaces ranging in size from 2 to 4,431 genes (up to 10% of the genome) were analyzed [9].

*Gentrepid *uses two modules: Common Pathway Scanning (CPS) and Common Module Profiling (CMP) to make candidate gene predictions.

The CPS module is based on the assumption that common phenotypes are associated with proteins that participate in the same protein complex or biochemical pathway [22]. Such systems biology methods are currently favored in candidate gene prediction because of the attractiveness of their basic thesis. Their weakness is the lack of coverage of the underlying systems biology knowledge bases [21]. Many tools attempt to ameliorate the deficits of the human systems biology knowledge base by extensive extrapolation of data from other species. Examples are GeneSeeker, ToppGene and Endeavour [13,23-25]. *Gentrepid *CPS uses only human data to reduce the number of predicted false positives i.e. it makes fewer predictions which are more often correct compared to other prediction systems [15].

The other module, CMP, is a novel sequence analysis approach based on the principle that candidate genes have similar functions to disease genes already determined for the phenotype [26]. *Gentrepid *CMP differs from most candidate gene prediction systems which describe functional similarity via keywords, a procedure which also lacks good coverage of the human genome [21]. In CMP, sequences are parsed at the domain level, linking them directly to function [21]. Although CMP's performance was disappointing in our original benchmark using a set of nine oligogenic diseases with Mendelian inheritance [12], it produced a surprising number of statistically significant results when confronted with the GWAS data on seven complex diseases [9]. This result was robust when compared with simulations using random SNPs, and may arise from an important underlying role for homologous genes in complex diseases.

## Drug-gene target data set

We compiled the drug-gene target data set from three publicly available drug databases: DrugBank [18], PharmGKB [19] and TTD [20]. Snapshots of these databases were taken in June 2012.

DrugBank is a freely available online database that combines detailed drug data and indication information with comprehensive drug-target associations [18]. From this database, we retrieved Drugbank IDs and drug names (generic and brand) to represent drugs, and the unique gene symbols to represent protein targets. We extracted 6,711 drug entries active against 3,410 unique drug targets from several species. We used the G-profiler conversion tool to separate human drug targets represented by official HUGO gene symbols [27], yielding 2,022 human drug targets associated with 3,910 drugs.

The Pharmacogenomics Knowledgebase (PharmGKB) is a drug knowledge base maintained by Stanford University, USA and funded by the US National Institute of Health (NIH). PharmGKB captures information about drugs, diseases/phenotypes and targeted genes [19]. From this database, we extracted the "drug-associated genes" field along with "description" which contains the disease information. This database contains around 3,097 drugs and 26,961 human genes, but not all these genes are associated with drugs. We retrieved 382 drugs for 566 human drug targets. For the PharmGKB database, the number of drug targets exceeds the number of drugs because some drugs target multiple genes.

The Therapeutic Target Database (TTD) is also a freely available online drug database which integrates drug data with therapeutic targets [20]. This database contains 17,816 drugs (approved, clinical and experimental) and 2,025 human and non-human (bacterial and fungal) drug targets. It describes synonyms of 3,167 drug names. We extracted "Drug names" along with "Disease" information, and "Uniprot accession numbers" for targets. UniProt accession numbers were replaced with official HUGO gene symbols using the G-profiler conversion tool [27]. Finally, we extracted 2,960 drugs for 544 unique human drug targets from TTD.

### Mapping of candidate gene data set with the drug-gene target data set

We mapped the list of 1,497 candidate genes with drug-gene target association files obtained from the three drug databases. The candidate genes for each disease were mapped with the three drug-target association files obtained from the three drug databases, and the results retrieved.

### Identification of novel therapeutics and therapeutic targets

In the next step, we identified novel therapeutic targets and therapeutics for all seven diseases. If an associated drug is not registered as a therapy for the phenotype of interest, it is predicted as a novel therapeutic for the new phenotype, directed towards the predicted candidate gene target. The novel drugs may be suitable for repositioning towards treatment of the phenotype in question.

### Validation of predicted therapeutic targets

The predicted therapeutic targets were validated using two benchmarks. In the first benchmark, the ability of the system to replicate known therapeutics *de novo *from the genetic data was assessed. This benchmark tests the system's ability to retrieve existing knowledge; however, this does not give any idea about the validity of the novel predictions. To test the system's ability for knowledge discovery, we performed an additional benchmark in which the validity of the candidate gene predictions for the phenotype were assessed using text mining of the literature.

In the first benchmark, genes present in the six search spaces were classified as "candidates" or "non-candidates". We considered genes which are currently known as drug targets for the phenotype of interest as "true positives". Targets with currently registered therapeutics for the phenotype of interest which were not predicted by *Gentrepid *but present in the search space were designated "false negatives". Genes which were not predicted and not targetable by drugs were "true negatives"; and, for the purpose of this benchmark, predicted novel therapeutic targets were considered "false positives". Receiver Operation Characteristic (ROC) Curves were plotted in GraphPad Prism 6 software considering six thresholds obtained from the number of targets present in the six search spaces for each phenotype. Linear, as well as non-linear regression analysis, was performed (see section Validation of predicted therapeutic targets in *Results and Discussion*).

In the second benchmark, all Pubmed IDs of literature related to Bipolar disorder, Type 1 diabetes, Type 2 diabetes, Crohn's disease, Coronary artery disease, Rheumatoid arthritis and Hypertension were extracted from Pubmed in Feb. 2013. For each target, we calculated the number of citations using both the gene name and the phenotype, by mapping the extracted Pubmed IDs to the gene citation information from Entrez Gene (ftp://ftp.ncbi.nih.gov/). Further, ROC curves were created in GraphPad Prism 6 software considering four thresholds of at least one, five, ten and fifteen citations. Non-linear regression analysis was also performed to fit the ROC curves (see section Validation of predicted therapeutic targets in *Results and Discussion*).

## Results and Discussion

### Comparison of drug databases

Firstly, we assessed coverage of the human genome by the three drug databases both individually and *in toto*. We extracted the following therapeutic drug-gene target association data from the three databases:

1. 3,910 drugs against 2,022 human targets from DrugBank [18];

2. 382 drugs against 566 human targets from PharmGKB [19] and;

3. 2,960 drugs against 544 human targets from TTD [20].

For more details about the content of these databases see *Methods*.

**Figure 2:**
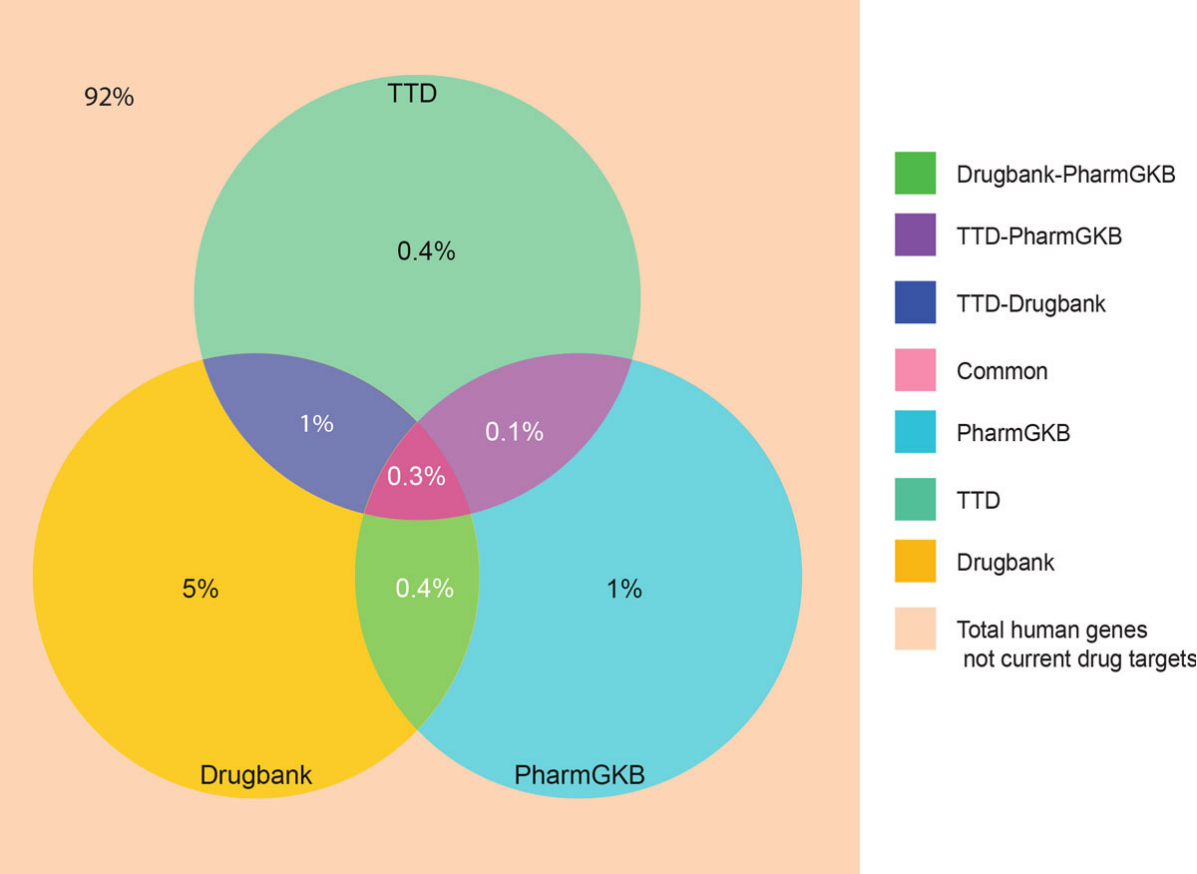
**Coverage of the human genome by targets annotated in the three drug databases**. The Venn diagram shows that gene targets annotated in drug databases comprise 8% of the entire human genome. It also describes the percentage of the genome covered by each database individually and upon pairwise comparison.

The total number of unique targets from all the databases was 2,494 genes, which is 8% of the entire human genome (Figure 2). Previously, it was estimated that 3,000-5,000 genes are druggable (able to be modulated by a small-molecule drug [28]) which is 10-17% of the entire genome [29-32]. The gap between extracted targets from the three drug databases (8%) and the estimated number of druggable genes (10-17%) exists because many druggable genes have not yet been mapped to a phenotype and thus there has been no imperative to develop drugs for these targets [33]. The targets searched in our study cover 50-83% of the possible druggable genes mentioned in previous studies [29-32].

**Figure 3:**
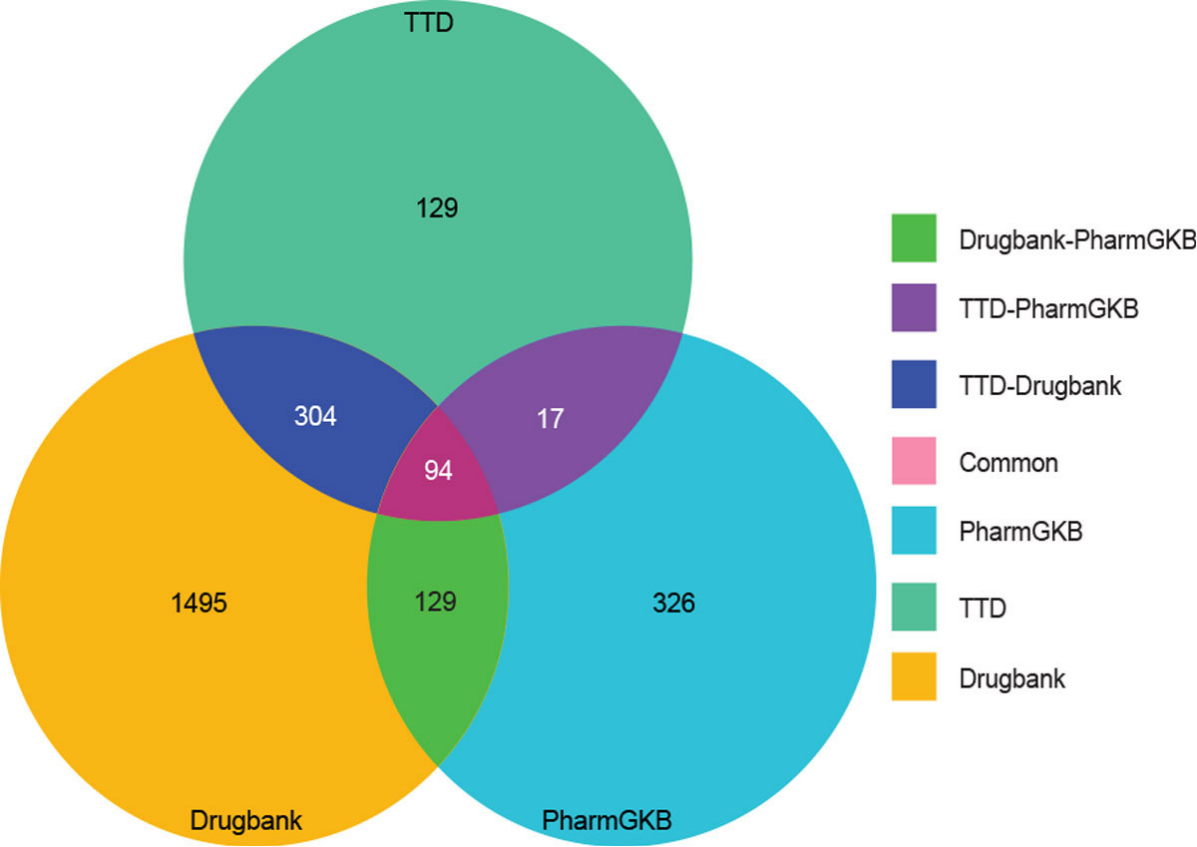
**Comparison of human drug targets from three drug databases**. Comparison of three drug databases to identify unique and common human drug targets extracted from DrugBank, TTD and PharmGKB. DrugBank has the highest number of unique human targets followed by PharmGKB and TTD.

We compared raw data such as drugs and drug targets across the three drug databases to determine the redundancy of the information in these databases. With respect to drug targets, only 4% of human drug target entries were common to all three databases (Figure 3). When the databases were compared in a pairwise fashion, the proportion of common targets ranged from 9-18%. Each of the databases contains a significant amount of information that is unique to that database. TTD has the fewest unique targets (129), while DrugBank and PharmGKB have 1,495 and 326 unique targets respectively (Figure 3).

**Figure 4:**
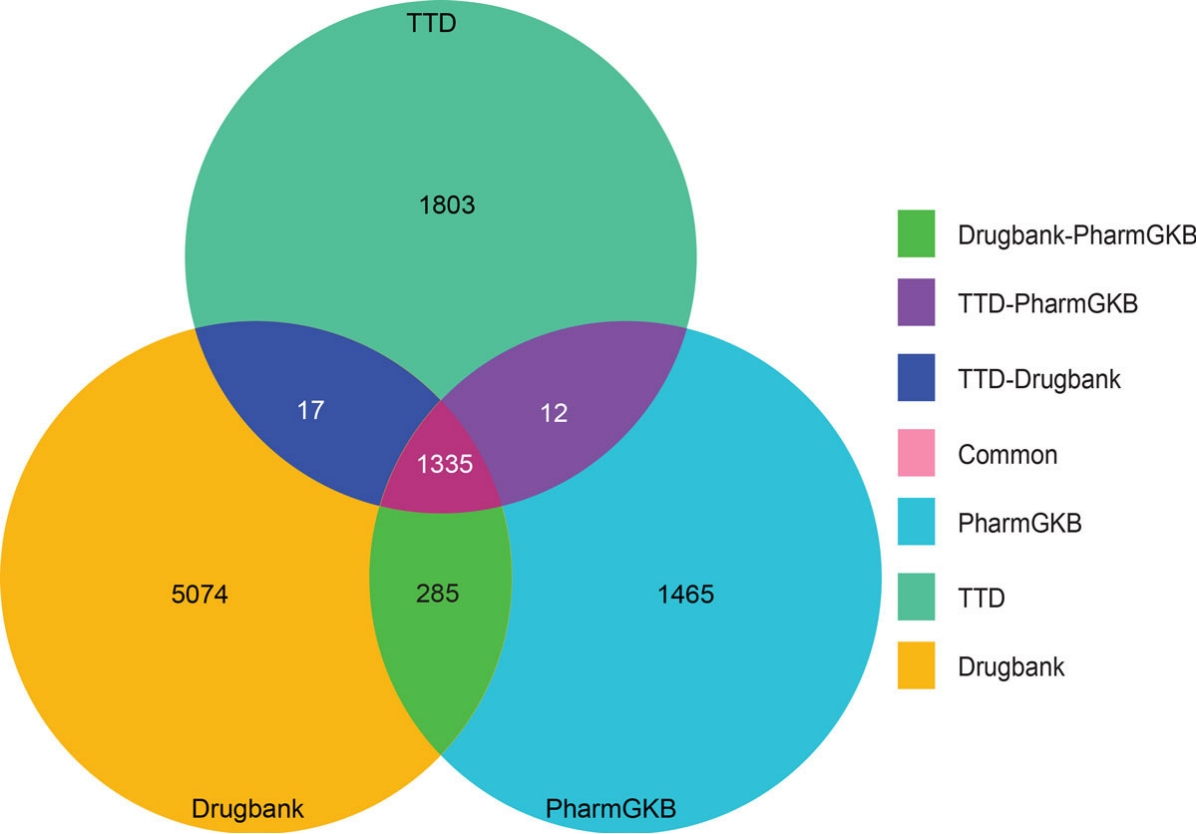
**Comparison of coverage of drugs in three drug databases**. Comparison of drug coverage of three drug databases to identify unique and common drugs. DrugBank has the highest number of unique drugs followed by TTD and PharmGKB.

We also compared the number of drugs present in three drug databases (Figure 4). Of the combined total of 9,991 unique drugs, DrugBank contributes 50% of the unique drug compounds, while TTD and PharmGKB, contribute 18% and 15% of the unique drug compounds respectively (Figure 4). Using pairwise comparisons to check redundancy of drugs between the databases, we observed TTD and PharmGKB share 15-19% of their listed drugs with DrugBank. Although there is significant overlap among the three databases, the high number of unique drugs in each database show the databases are fairly complementary. In summary, all three drug databases contain unique and valuable data and were thus all used in the subsequent analysis.

## Identification of therapeutic targets

**Figure 5:**
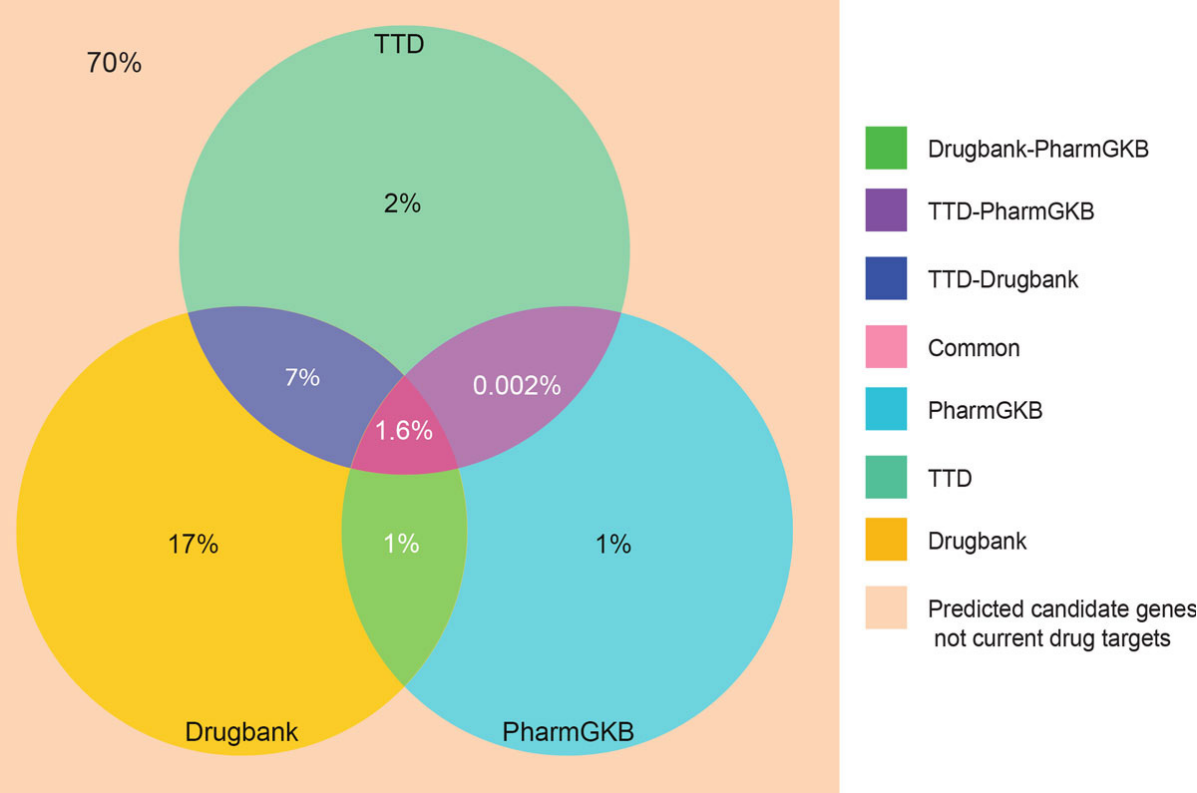
**Predicted therapeutic targets from three source databases**. The Venn diagram represents the identified 30% of 1,497 candidates are potential therapeutic targets for all the seven diseases. 17% of the targets were unique to one of the three drug databases (DrugBank), 1-2% of targets were found in at least two databases (PharmGKB, TTD) and only 1.6% of targets are common to all the three drug databases.

We identified potential therapeutic targets for the seven complex diseases from the *Gentrepid *predicted candidate genes generated by our reanalysis of the WTCCC data. In total, *Gentrepid *predicted 1,497 candidate genes for all seven diseases; comprising by phenotype: Type 2 Diabetes (291), Bipolar Disorder (212), Crohn's Disease (378), Hypertension (219), Type 1 Diabetes (358), Coronary Artery Disease (264) and Rheumatoid Arthritis (200) (Additional file 1 Table S1) [9]. We searched for these candidate genes in the drug-gene target files obtained from all three drug databases and found 452 potential therapeutic targets for the seven complex diseases (Table 1). This illustrates that almost 30% of the total number of predicted candidate genes by *Gentrepid *are potential targets for therapeutic treatments using currently available drugs (Figure 5). The disparity between the 8% of the human genome that is targettable (2,494 extracted targets - Figure 2) and the 30% of predicted candidate genes that are targettable (452 predicted targets - Figure 5) is interesting and should be investigated further. The enrichment of druggable targets in the candidate gene set might be a selection effect: either at the SNP level; or at the knowledgebase level: it might suggest that we already know more about disease genes than the genome in general. Alternatively, it has been previously suggested that the genome can be partitioned into "disease genes" and "non-disease genes". While such a Boolean distribution is likely to be overly simplistic, a spectrum of levels of disease association with specific gene subsets might explain this disparity.

**Table 1:** Repositioning potential and known therapeutic targets by phenotype.

PH	*≠ TT*	TI	RN	RTT	NTT	NV	RN
T2D	84	0.29	5^th^	7	77	0.92	5^th^
T1D	97	0.27	6^th^	2	95	0.98	2^nd^
RA	77	0.38	2^nd^	6	71	0.92	5^th^
HT	78	0.35	4^th^	5	73	0.94	4^th^
BD	59	0.27	6^th^	1	58	0.98	2^nd^
CD	135	0.36	3^rd^	0	135	1.00	1^st^
CAD	102	0.39	1^st^	4	98	0.96	3^rd^

Abbreviations - PH - Phenotypes; ≠TT - Number of Therapeutic Targets; TI - Targetability Index; NTT - Novel Therapeutic Targets; RTT - Replicated Therapeutic Targets; NV - Novelty; RN - Rank; T2D - Type 2 Diabetes; BD - Bipolar Disorder; CD - Crohn's Disease; HT - Hypertension; T1D - Type 1 Diabetes; CAD - Coronary Artery Disease; RA - Rheumatoid Arthritis.

Description of therapeutic targets and novel therapeutic targets. Total 452 unique therapeutic targets and total 428 unique novel therapeutic targets obtained for seven complex diseas

To drill a little further into the data, we assessed the therapeutic potential of each phenotype using currently available repositioned drugs. We calculated an empirical Targetability Index (TI), defined here as the ratio of the number of predicted targets to the number of predicted candidate genes for each phenotype (Table 1). The distribution was bimodal with four phenotypes (CAD > RA > CD > HT) being more targetable (TI = 0.35-0.39) than the other three (T2D > T1D ~ BD) (TI = 0.27-0.29). A factor which is likely to influence the targetability is our underlying knowledge of the phenotype. If the molecular pathways involved have been previously characterized, there is more likely to be drug-target information in the existing drug databases, even if the phenotype has not previously been associated with the molecular system. The low TIs for BD (0.28) and the diabetes phenotypes (0.27-0.29) likely arises from lack of knowledge of underlying pathways. More basic research in this area is required.

All three drug databases made significant contributions to target identification, with the highest contribution from DrugBank (400), followed by TTD (156) and PharmGKB (61). DrugBank is a chemical as well as a clinical drug database which contains broader coverage of drug targets and broader depth of information compared to the chemical drug database TTD and the clinical drug database PharmGKB. PharmGKB, being a clinical drug database, has a lower coverage of drug-target associations, but broader depth of information compared to TTD. To summarize, the total coverage of the predicted targets from all three databases was estimated to be 30% of the candidate genes predicted by *Gentrepid*, with the maximum contribution from DrugBank (Figure 5).

## Discovery of novel therapeutic targets

For the seven diseases considered in our study, we performed a binary classification of the 452 targets to distinguish therapeutic targets which were "rediscovered" (or replicated) from novel potential therapeutic targets. Novel genes are targeted by therapeutics registered for other uses but not for the phenotype of interest. We found 428 novel therapeutic targets accounting for almost 94% of the targets identified in the previous section. The remaining 24 targets have therapeutics which either are approved, are in ongoing clinical trials, or have been discontinued as therapeutics for the phenotype of interest (Table 2). We considered these 24 known targets as "true positives" for the phenotypes of interest in one of the benchmarks described below (see section Validation of predicted therapeutic targets in *Results and discussion*).

**Table 2:** Predicted known therapeutics

PH	Target	*Drug name	Status	Action	*Database
T1D	*PPARG*	Rosiglitazone	Approved	Agonist	TTD
	*DGKA*	Vitamin E	Approved	Unknown	DrugBank

T2D	*CTSD*	Insulin Regular	Approved	Unknown	DrugBank
	*PPARA*	Aleglitazar	Phase III	Agonist	TTD
	*NR3C1*	ISIS-GCCR	Preclinical	Antisense	TTD
	*TCF7L2*	Repaglinide	Unknown	Unknown	PharmGKB
	*PPARD*	Bezafibrate	Approved	Agonist	DrugBank
	*RB1*	Insulin, porcine	Approved	Unknown	DrugBank
	*HSD11B1*	INCB13739	Phase IIa	Inhibitor	TTD

RA	*TNF*	Infliximab	Approved	Inhibitor	DrugBank
	*ITGA4*	CDP323	Phase II	Antagonist	TTD
	*JAK2*	INCB18424	Phase III	Inhibitor	TTD
	*IL15*	AMG-714	Discontinued in phase I	Inhibitor	TTD
	*CCL2*	MCP-1	Preclinical	Inhibitor	TTD
	*PRKCA*	Vitamin E	Approved	Unknown	DrugBank

HT	*DRD1*	Fenoldopam	Approved	Agonist	TTD
	*AGTR1*	Valsartan	Approved	Antagonist	TTD
	*CNR1*	AZD1175	Discontinued in phase I	Antagonist	TTD
	*AGT*	Benazepril	Unknown	Unknown	PharmGKB
	*GUCY1A2*	Isosorbide Mononitrate	Approved	Inducer	DrugBank

BD	*SLC6A2*	Imipramine	Approved	Inhibitor	DrugBank
	*AGTR1*	Valsartan	Approved	Antagonist	DrugBank

CAD	*MYC*	AVI4126	Phase I/II	Antisense	TTD
	*PLG*	Urokinase	Approved	Activator	DrugBank
	*NOS3*	ACCLAIM	Phase III	Stimulator	TTD

Abbreviations - PH - Phenotypes; T2D - Type 2 Diabetes; BD - Bipolar Disorder; HT - Hypertension; T1D - Type 1 Diabetes; CAD - Coronary Artery Disease; RA - Rheumatoid Arthritis; TTD - Therapeutic Target Database; PharmGKB - Pharmacogenomics Knowledgebase.

Therapeutic targets with predicted known therapeutics for phenotypes of interest.

(^* ^Drugs mentioned in the table are only examples as one target may have multiple drugs);

(^* ^Drug databases in the table are only examples as one drug-target association may be present in more than one database).

**Figure 6:**
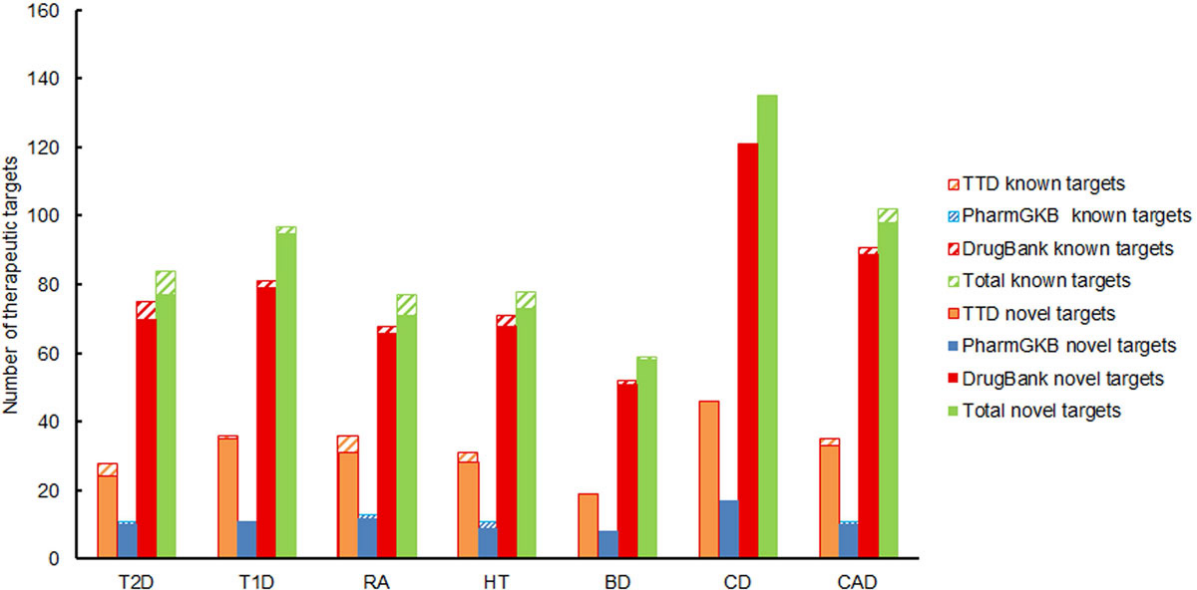
**Predicted therapeutic targets for each of the seven phenotypes**. Abbreviations - T2D - Type 2 Diabetes; BD - Bipolar Disorder; CD - Crohn's Disease; HT - Hypertension; T1D - Type 1 Diabetes; CAD - Coronary Artery Disease; RA - Rheumatoid Arthritis. For each phenotype, the contributions of each of the three drug databases are shown in primary colours on the left, and the set of total unique targets is shown in green on the right. The cross-hatched portion of the bar shows targets replicated by the system which are already targeted by therapeutics for that phenotype. The solid portions of the bars are novel predictions, which may potentially be utilized in repositioning.

Figure 6 shows the number of novel therapeutic targets obtained for each of the seven diseases, along with the contribution from each drug database. The novelty of the predicted targets for each disease was assessed by calculating the ratio of the number of novel therapeutic targets to the number of therapeutic targets predicted for each disease. The novelty ratio for all diseases was between 0.92 and 1.0 (Table 1). We observed the highest novelty ratio for CD (1.0) and the lowest for RA (0.92). The high ratio of novel targets for all phenotypes to predicted targets suggests repositioning could have a large impact on clinical studies.

## Identification of novel therapeutics

To identify novel drugs, we compared our phenotype of interest (from the pool of seven diseases considered in our study) with indications associated with the drug. In total, we retrieved 7,252 drugs associated with human drug targets from all three drug databases. We found 2,192 (30% of the extracted drugs) unique drugs that target the 452 potential therapeutic targets.

We retrieved the maximum number of drugs from DrugBank (1,618) and the remainder from TTD (735) and PharmGKB (91). In order to identify the novel drugs i.e. drugs not targeting our phenotype of interest, we filtered the above list of 2,192 drugs to retrieve 2,130 novel therapeutics. On a phenotype by phenotype basis, T1D and CAD had the maximum number of novel predicted therapeutics. Although CD had the highest number of novel targets, it had comparatively few novel therapeutics suggesting new drug development is needed for this phenotype. BD had the fewest therapeutics as expected based on the small number of predicted therapeutic targets. We found that the total percentage of drugs that may be repositioned towards identified novel targets was around 29% of the total number of extracted drugs.

Table 2 shows the 24 replicated targets with examples of replicated drugs found in our study. For example, the drug "Aleglitazar" is in phase III clinical trial for the T2D target *PPARA*, a predicted candidate gene for T2D. "Rosiglitazone" known to target *PPARG *as a therapeutic for diabetes mellitus, has a potential use in the related phenotype T1D.

Examples of novel therapeutics for the seven phenotypes are shown in Table 3. For example, "Pirenzepine", which acts upon the *CHRM1 *gene product, is approved as a therapeutic drug for peptic ulcers. Our study predicts *CHRM1 *is a predicted candidate gene and novel therapeutic target for T2D, suggesting that the drug Pirenzepine may be repositioned as a novel therapeutic for T2D. Hence, the associated therapeutics for the novel therapeutic targets may be repositioned against the phenotypes of interest, accelerating the drug discovery process.

**Table 3:** Novel therapeutics suitable for repositioning for the seven diseases

PH	Target	*Drug name	Status	Current Indication	Action	*Database
T1D	*RARA*	Alitretinoin	Approved	Kaposi's sarcoma	Agonist	TTD
	*GSK3B*	Lithium	Unknown	Bipolar disorder	Unknown	PharmGKB

T2D	*CHRM1*	Pirenzepine	Approved	Peptic ulcer disease	Antagonist	TTD
	*LPL*	Gemfibrozil	Approved	Hyperlipidemia	Activator	TTD

CAD	*FLT1*	Sorafenib	Launched	Advanced renal cell carcinoma	Inhibitor	TTD
	*KDR*	Sunitinib	Launched	Advanced renal cell carcinoma	Inhibitor	TTD

BD	*ESR1*	Trilostane	Approved	Cushing's syndrome	^a^Modulator	DrugBank
	*ABCC1*	Methotrexate	Unknown	Psoriasis	Unknown	PharmGKB

HT	*TACR1*	GSK1144814	Phase I	Schizophrenia	Antagonist	TTD
	*NRP1 *	Palifermin	Approved	Oral mucositis	Unknown	DrugBank

CD	*CRHR1*	CRF-1 antagonist	Phase II completed	Irritable bowel syndrome	Antagonist	TTD
	*INSR*	Insulin Detemir	Approved	Type I and II Diabetes	Agonist	DrugBank

RA	*HLA - DRB 1*	Glatiramer Acetate	Approved	Multiple sclerosis	Binder	TTD
	*ACE*	Ramipril	Approved	Hypertension	Inhibitor	DrugBank

Abbreviations - PH - Phenotypes; T2D - Type 2 Diabetes; T1D - Type 1 Diabetes; CAD - Coronary Artery Disease; BD - Bipolar Disorder; HT - Hypertension; CD - Crohn's Disease; RA - Rheumatoid Arthritis; TTD - Therapeutic Target Database; PharmGKB - Pharmacogenomics Knowledgebase.

Examples of novel therapeutics suitable for repositioning towards cure of seven diseases. (* Drugs mentioned in the table are only examples as one target may have multiple drugs); (* Drug databases in the table are only examples as one drug-target association may be present in more than one database); (^a ^Allosteric Modulator).

### FDA-approved and clinical targets

**Figure 7:**
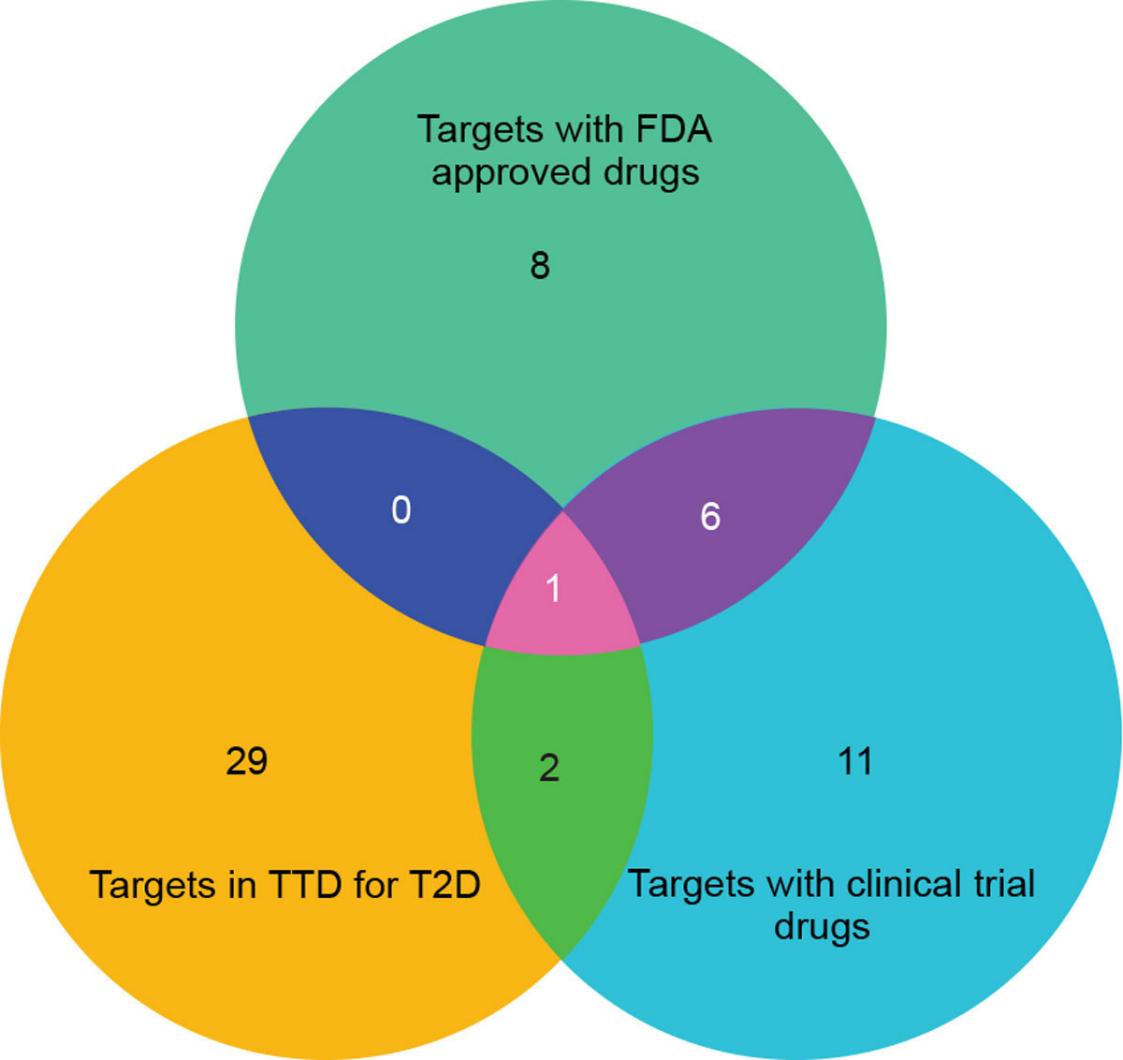
**FDA-approved and clinical therapeutic targets**. Abbreviation - T2D - Type 2 Diabetes; Comparison of *Gentrepid *predicted targets for Type 2 diabetes targeted by FDA-approved and clinical trial drugs with targets obtained from the TTD database for Type 2 Diabetes. Three predicted therapeutic targets (*HSD11B1, PPARA, NR3C1*) targeted by drugs currently in clinical trials for T2D. In addition, *PPARA *is also targeted by FDA-approved drugs.

Identification of therapeutic targets targeted by approved and clinical trial drugs can help us to prioritize drugs for repositioning against phenotypes of interest. Both approved and clinical targets are potential drug targets, however, approved targets will undoubtedly be on the priority list for further experimental studies. We classified the predicted targets as FDA-approved and clinical targets for the seven complex diseases. An example depicted in Figure 7 shows comparison between T2D targets from the TTD database and targets predicted by *Gentrepid *for T2D. Of the 84 targets predicted for T2D by *Gentrepid *(Table 1), 28 are listed in TTD (Figure 7). Comparing these 28 targets with the 32 targets indicated for T2D in TTD, we found products of three genes (*HSD11B1, PPARA, NR3C1*) are targeted by drugs currently in clinical trials for T2D. In addition, *PPARA *is already targeted by FDA-approved drugs. Hence, we predicted 25 novel therapeutic targets from the TTD database for T2D. In total for the seven diseases, we found 291 approved therapeutic targets and 95% of these as novel approved targets. We also found 334 targets in clinical trials and 96% of these being novel (Table 4). To summarize, both approved and clinical novel targets are associated with therapeutics, which may be repositioned as novel treatments towards the cure of complex diseases.

**Table 4:** Approved and clinical targets for seven complex diseases

PH	AT	NAT	CT	NCT
T2D	45	41	65	62
T1D	57	55	73	72
HT	71	68	43	40
RA	55	53	59	54
CD	93	93	135	135
CAD	63	61	80	76
BD	37	36	44	44
Unique sum	291	277	334	318

Abbreviations - PH - Phenotypes; AT - Approved Targets; NAT - Novel Approved Targets; CT - Clinical Targets; NCT - Novel Clinical Targets; T2D - Type 2 Diabetes; T1D - Type 1 Diabetes; HT - Hypertension; RA - Rheumatoid Arthritis; CD - Crohn's Disease; CAD - Coronary Artery Disease; BD - Bipolar Disorder.

Predicted therapeutic targets targeted by FDA-approved drugs and drugs in clinical trials.

### Validation of predicted therapeutic targets

To assess the validity of targets predicted by *Gentrepid *for each phenotype, we used two different benchmarks. In the first benchmark, validity of the association of the gene with the phenotype was based on whether they are designated as targets in the drug databases or not. This was repeated for all six search spaces investigated for each phenotype. In the second benchmark, the validity of the association of the gene with the phenotype was assessed by the existence or the absence of abstracts in the literature citing both the gene name and the phenotype.

For the first benchmark, we performed a binary classification of genes in the six search spaces as "candidates" or "non-candidates". As described in Table 5, targets with therapeutic drugs for the phenotype of interest were considered "true positives". Targets with currently registered therapeutics for the phenotype of interest which were not predicted by *Gentrepid*, but were present in the search space as "false negatives". Genes which were not predicted and not targetable by drugs as "true negatives" and *Gentrepid*-predicted novel therapeutic targets were considered as "false positives" (Table 5). ROC curves were plotted considering targets based on the six search spaces from the weakly significant data set (Additional file 1, Figure S1). Area Under Curve (AUC) values obtained from these ROC curves were significantly greater than 0.5 (p < 0.05) (Additional file 1, Table S2). This suggests that our predictions of novel therapeutic targets for all the seven diseases are significant.

**Table 5:** Binary classification of therapeutic targets

PH	Total genes in all search spaces	Binary classification	
T2D	4,292	TP = 7	FP = 77
		FN = 9	TN = 4,199

T1D	5,339	TP = 2	FP = 95
		FN = 9	TN = 5,233

HT	8,427	TP = 5	FP = 73
		FN = 15	TN = 8,334

RA	4,970	TP = 6	FP = 71
		FN = 9	TN = 4,884

BD	5,667	TP = 1	FP = 58
		FN = 6	TN = 5,602

CD	5,644	TP = 0	FP = 135
		FN = 0	TN = 5,509

CAD	4,715	TP = 4	FP = 98
		FN = 8	TN = 4,605

Abbreviations - TP - True Positives; FP - False Positives; TN - True Negatives; FN - False Negatives; TN - True Negatives; PH - Phenotypes; T2D - Type 2 Diabetes; T1D - Type 1 Diabetes; HT - Hypertension; RA - Rheumatoid Arthritis; CD - Crohn's Disease; CAD - Coronary Artery Disease; BD - Bipolar Disorder.

Binary classification of therapeutic targets considering targets present in six search spaces from weakly significant data set.

For the second benchmark, ROC curves for the seven complex diseases were created by considering four thresholds for targets cited by at least one, five, ten and fifteen article citations as true positives and targets without any citations or less than five, ten and fifteen citations as false positives. Figure S2 (Additional file 1) contains all the ROC curves and Table S2 (Additional file 1) contains the AUC values. The AUC values for all the seven diseases were significantly greater than from 0.5 (p < 0.05) meaning that our results were significantly better than by chance. This also suggests that our predictions of novel therapeutic targets for all seven diseases are significant.

## Significance of the work

The primary purpose of our work was to identify potential therapeutics and their targets by integrating publicly available genetic, bioinformatic and drug data using the *Gentrepid *candidate gene prediction platform. As the method involves repositioning of currently available drugs, it allows immediate translational opportunities for drug testing [8]. Other bioinformatic tools have been used to identify potential therapeutic targets for complex diseases and other conditions. For example, TARGET gene was used to identify and prioritize potential targets from hundreds of candidate genes for different types of cancer [34]. Another study identified potential drug targets for three neurological disorders - Alzheimer's disease, Parkinson's disease and Schizophrenia. This study involved the prediction of candidate genes using the ToppGene and ToppNet prediction systems [24,35]. The repositioning tools could be used as an initial screening tool for potential drugs which can be used for further evaluation [34]. It is important to note that not all repositioning opportunities will be successful as there are always some limitations [36,37].

## Conclusion

There is a need to develop new approaches for the identification of therapeutic targets to accelerate the process of therapeutic drug discovery which has not kept pace with discoveries in genetics. In this study, we integrated detailed drug data with predicted candidate genes for seven complex diseases. We found 29% of the predicted candidate genes could serve as novel therapeutic targets and 29% of the extracted drugs are potential novel therapeutics for at least one of the seven complex diseases considered in our study. We have utilized both FDA-approved drugs and drugs in clinical trials. Further investigation is required to verify the action of these drugs. This study enables efficient identification of possible novel therapeutic targets and alternative indications for existing therapeutics. Hence, these drugs may be repositioned against seven phenotypes of interest, quickly taking advantage of already done work in pharmaceutics to translate ground-breaking results in genetics to clinical treatments. *Gentrepid*, thus can be utilized as a drug screening tool to save time and money spent on the initial stages of drug discovery.

## List of abbreviations

GWAS: Genome-Wide Association Study; WTCCC: Wellcome Trust Case-Control Consortium; EMEA: European Medicinal Agency; FDA: Food and Drug Administration; SNP: Single Nucleotide Polymorphism; CPS: Common Pathway Scanning; CMP: Common Module Profiling; BD: Bipolar Disorder; CAD: Coronary Artery Disease; CD: Crohn's Disease; HT: Hypertension; RA: Rheumatoid Arthritis; T1D: Type 1 Diabetes; T2D: Type 2 Diabetes; WS: Weakly Significant set; MWS: Moderately-Weak Significant set; MHS: Moderately-High Significant set; HS: Highly Significant set; TTD: Therapeutic Target Database; PharmGKB: Pharmacogenomics Knowledgebase; AUC: Area Under Curve; PH: Phenotypes; TI: Targetability Index; ROC: Receiver Operation Characteristic curve.

## Competing interests

The authors have declared that no competing interests exist.

## Authors' contributions

MPG carried out the data mining and analysis, and worked on the design of the project. MAW conceived the study, participated in its design and reviewed the results from the data analysis. MPG, MAW, TMC, KAM, CDS, SB and RAG helped to draft the manuscript. All authors read and approved the final manuscript.

## Supplementary Material

Additional file 1 **Gentrepid annotated SNPs, ROC curves and AUC values for seven phenotypes**. Table S1 - Gentrepid annotated SNPs (clusters) for four data sets, WTCCC study associated loci (HS - p ≤ 5 x10^-7 ^) and Gentrepid predicted candidate genes per phenotype. Figure S1 - ROC curves for seven diseases based on six thresholds obtained from targets present in six search spaces in weakly significant data set. Table S2 AUC values for ROC curves. Figure S2 ROC curves for seven diseases based on four thresholds obtained using four cutoff of Pubmed citations (at least one, five, ten and fifteen). Click here for file

## Declarations

The publication costs for this article were funded by the corresponding author (MAW).

This article has been published as part of *BMC Medical Genomics *Volume 7 Supplement 1, 2014: Selected articles from the 3rd Translational Bioinformatics Conference (TBC/ISCB-Asia 2013). The full contents of the supplement are available online at http://www.biomedcentral.com/bmcmedgenomics/supplements/7/S1.
