# Supplementary material for: Identification of novel therapeutics for complex diseases from genome-wide association data
Source: BMC Med Genomics. 2014 May 8;7(Suppl 1):S8. doi: 10.1186/1755-8794-7-S1-S8 (PMC4101352; doi:10.1186/1755-8794-7-S1-S8)
Supplement: Additional file 1 — Gentrepid annotated SNPs, ROC curves and AUC values for seven phenotypes. Table S1 - Gentrepid annotated SNPs (clusters) for four data sets, WTCCC study associated loci (HS - p ≤ 5 x10^-7^ ) and Gentrepid predicted candidate genes per phenotype. Figure S1 - ROC curves for seven diseases based on six thresholds obtained from targets present in six search spaces in weakly significant data set. Table S2 AUC values for ROC curves. Figure S2 ROC curves for seven diseases based on four thresholds obtained using four cutoff of Pubmed citations (at least one, five, ten and fifteen). [file 1755-8794-7-S1-S8-S1.pdf]

Table S1 - SNPs per phenotype, WTCCC study associated loci (HS) and *Gentrepid* predicted candidate genes per phenotype

Phenotype	WS $p \leq 1x10^{-3}$ SNPs (Clusters)	MWS $p \leq 1x10^{-4}$ SNPs (Clusters)	MHS $p \leq 1x10^{-5}$ SNPs (Clusters)	HS $p \leq 5x10^{-7}$ SNPs (Clusters)	Loci in the original WTCCC Study	*Gentrepid* predicted candidate genes
CAD	757 (436)	134 (86)	43 (22)	25 (10)	1	264
HT	799 (460)	110 (61)	5 (5)	0 (0)	0	219
T2D	725 (421)	125 (70)	45 (15)	19 (2)	3	291
T1D	966 (442)	276 (103)	162 (43)	92 (24)	7	358
RA	699 (429)	104 (75)	27 (14)	11 (5)	3	200
CD	1064 (501)	261 (112)	102 (23)	63 (10)	9	378
BD	797 (513)	138 (94)	23 (10)	0 (0)	1	212

Abbreviations - T2D - Type 2 Diabetes; BD - Bipolar Disorder; CD - Crohn's Disease; HT - Hypertension; T1D - Type 1 Diabetes; CAD - Coronary Artery Disease; RA - Rheumatoid Arthritis; SNP - Single Nucleotide Polymorphism; WTCCC - Wellcome Trust Case-Control Consortium; WS: Weakly Significant Set; MWS: Moderately-Weak Significant Set; MHS: Moderately-High Significant set; HS: Highly Significant Set.

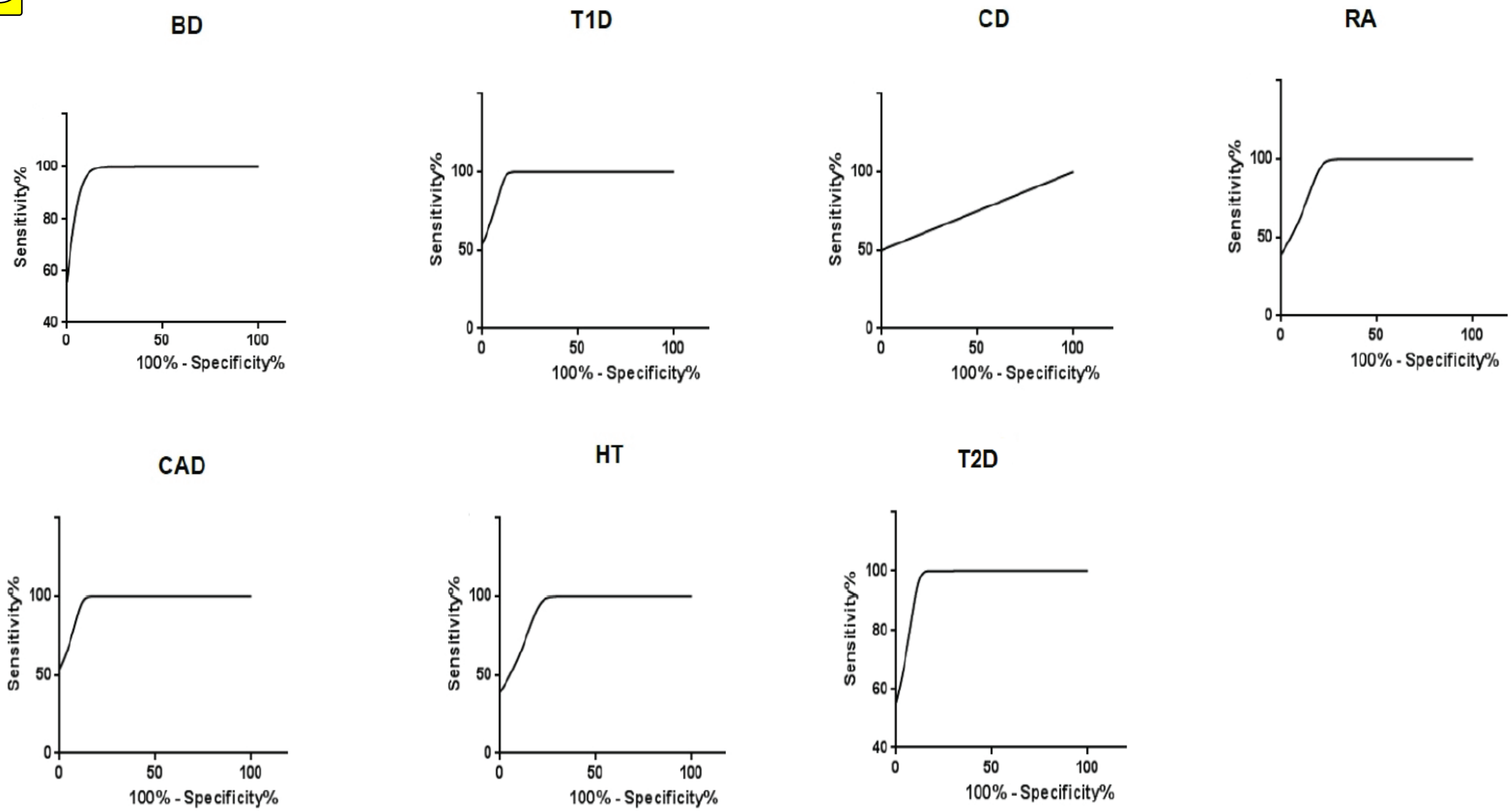
BD
Sensitivity%
100
80
60
40
0
50
100
100% - Specificity%
T1D
Sensitivity%
100
50
0
0
50
100
100% - Specificity%
CD
Sensitivity%
100
50
0
0
50
100
100% - Specificity%
RA
Sensitivity%
100
50
0
0
50
100
100% - Specificity%
CAD
Sensitivity%
100
50
0
0
50
100
100% - Specificity%
HT
Sensitivity%
100
50
0
0
50
100
100% - Specificity%
T2D
Sensitivity%
100
80
60
40
0
50
100
100% - Specificity%

Table S2 - AUC values for ROC curves

Phenotype	AUC (figure S1)	AUC (figure S2)
T2D	0.98	0.95
T1D	0.97	0.97
RA	0.95	0.97
HT	0.95	0.97
BD	0.98	0.98
CD	0.99	0.97
CAD	0.97	0.97

Abbreviations - T2D - Type 2 Diabetes; BD - Bipolar Disorder; CD - Crohn’s Disease; HT - Hypertension; T1D - Type 1 Diabetes; CAD - Coronary Artery Disease; RA - Rheumatoid Arthritis; AUC - Area under curve.

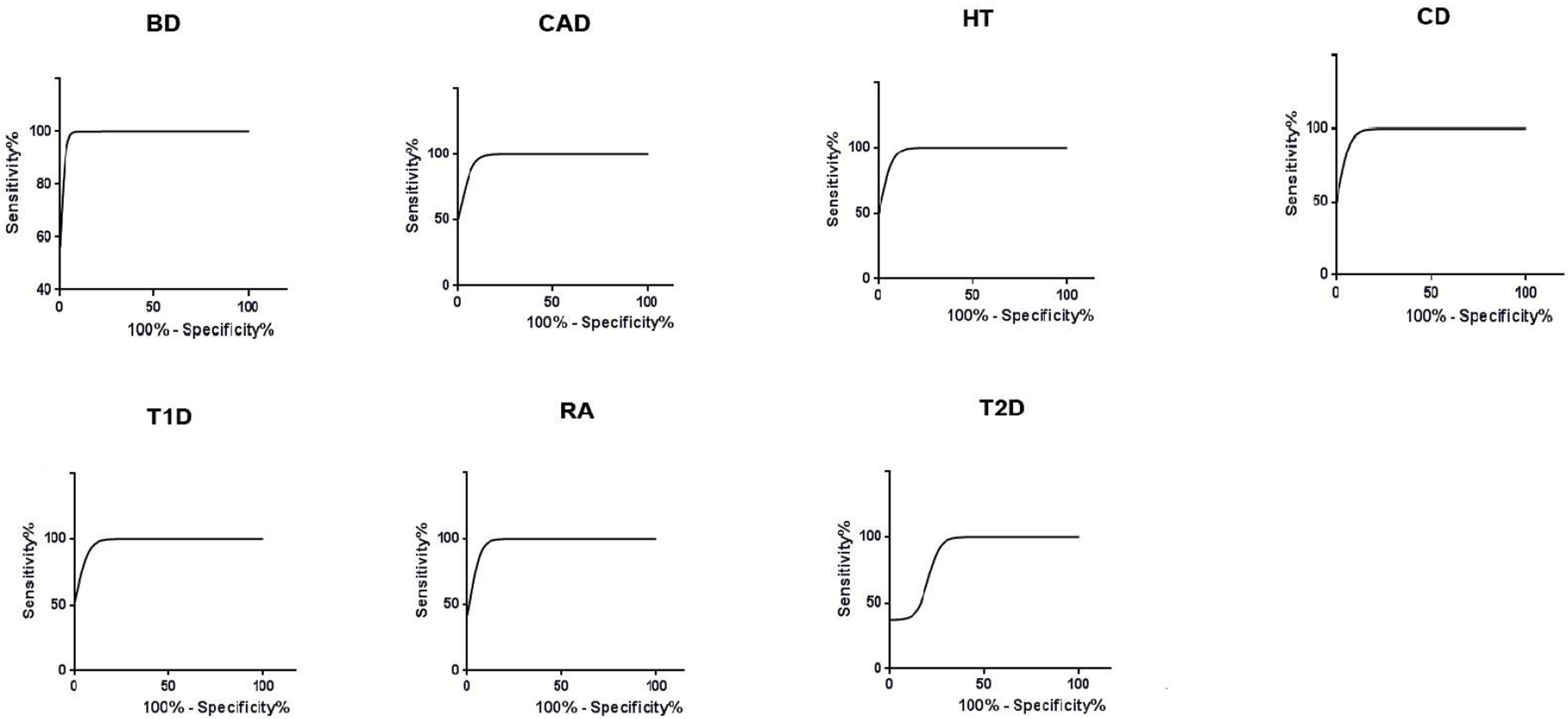
BD
Sensitivity%
100
80
60
40
0
50
100
100% - Specificity%
CAD
Sensitivity%
100
50
0
0
50
100
100% - Specificity%
HT
Sensitivity%
100
50
0
0
50
100
100% - Specificity%
CD
Sensitivity%
100
50
0
0
50
100
100% - Specificity%
T1D
Sensitivity%
100
50
0
0
50
100
100% - Specificity%
RA
Sensitivity%
100
50
0
0
50
100
100% - Specificity%
T2D
Sensitivity%
100
50
0
0
50
100
100% - Specificity%
